# Cemented versus Cementless Total Hip Arthroplasty for Femoral Head Osteonecrosis: A Study Based on National Claim Data in South Korea

**DOI:** 10.5435/JAAOSGlobal-D-23-00029

**Published:** 2023-05-02

**Authors:** Jung-Wee Park, Young-Seung Ko, Sojeong Park, Sung Hwa Kim, Young-Kyun Lee, Kyung-Hoi Koo

**Affiliations:** From the Department of Orthopaedic Surgery, Seoul National University Bundang Hospital, Seongnam, South Korea (Dr. J-W. Park, Dr. Ko, Dr. Lee, and Dr. Koo); the Data Science Team, Hanmi Pharm. Co., Ltd, Seoul, Korea (Ms. S. Park); the Department of Biostatistics, Yonsei University Wonju College of Medicine, Wonju, Korea (Mr. Kim); the Department of Orthopaedic Surgery, Seoul National University College of Medicine, Seoul, South Korea (Dr. Lee); and the Department of Orthopaedic Surgery, Kay Joint Center, Cheil Orthopaedic Hospital, Seoul, South Korea (Dr. Koo).

## Abstract

The purpose of this study was to compare the rates of revision, periprosthetic joint infection (PJI), and periprosthetic fracture (PPF) between patients with osteonecrosis of the femoral head (ONFH) undergoing noncemented total hip arthroplasty (THA) and cemented THA using a national claim data in South Korea.

**Methods:**

We identified patients who received THA for ONFH from January 2007 to December 2018 using *ICD* diagnosis codes and procedural codes. Patients were categorized into two groups according to the fixation method: with or without cement. The survivorship of THA was calculated using the following end points: revision of both the cup and stem, revision of the single component, any type of revision, PJI, and PPF.

**Results:**

A total of 40,606 patients: 3,738 patients (9.2%) with cement and 36,868 patients (90.7%) without cement, received THA for ONFH. The mean age of the noncemented fixation group (56.2 ± 13.2 years) was significantly lower than that of the cemented fixation group (57.0 ± 15.7 years, *P* = 0.003). The risk of revision and PJI was notably higher in cemented THA (hazard ratio: 1.44 [1.21 to 1.72] and 1.66 [1.36 to 2.04], respectively). Noncemented THA had a higher 12-year survivorship compared with cemented THA with any revision and PJI as the end point.

**Discussion:**

Noncemented fixation had better survivorship than cemented fixation in patients with ONFH.

Most patients regain pain relief and good functional outcome after total hip arthroplasty (THA) irrespective of the fixation method, with or without cement.^1-3^ However, cardiopulmonary complication is a serious risk of cemented fixation,^4,5^ whereas there is a higher incidence of periprosthetic fracture when using noncemented stems.^6,7,8,9^ Thus, using or not using cement for implant fixation has been a controversial issue of THA during the past several decades.

A number of studies have compared cemented and noncemented fixations.^2,6,7,8,9,10,11,12,13,14^ A few studies reported comparable or better functional results and survival of noncemented THA compared with cemented THA,^2,9,13,15-17^ whereas in other studies, cemented stems performed better in restoring function and pain relief with fewer complications.^6-8,18^ Most studies supporting the use of cement were performed in elderly patients, and the follow-up period was short. In those studies, less than 50% of the patients were available at 5 to 10 years postoperatively.

Osteonecrosis of the femoral head (ONFH) affects young and middle-aged adults, who demand high activity and have a long life expectancy. In this national medical claim data study, we compared the rates of revision, periprosthetic joint infection (PJI), and periprosthetic fracture (PPF) between patients with ONFH undergoing noncemented THA and those undergoing THA with the use of cement.

## Methods

### Database

This study was conducted as a registry-based study of patients who were treated with THA for ONFH in South Korea using the Korean Health Insurance Review and Assessment (HIRA) database. HIRA receives the entire South Korean medical claims data through the Korea National Health Insurance Program, which covers 97% of the South Korean cohort. Medical aid program supported by the South Korean government covers the remaining 3%. In the HIRA database, the patient characteristics, diagnoses, and procedures are classified according to the *International Classification of Diseases, Tenth Revision* (*ICD-10*) codes and Electronic Data Interchange (EDI) codes.

### Patient Identification

In January 2022, we identified patients who received THA for ONFH from January 2007 to December 2018 in South Korea using *ICD* diagnosis codes of ONFH (M8705, M8715, M8725, M8735, M8785, M8795, M9035, M9045, and M9055) and then the procedural codes of THA (N0711 and N2070). Because studies involving bilateral cases can bias results,^19^ we did not include cases of bilateral THA. As the laterality of the operated hip was not coded in the HIRA database, patients who received THA two times or more during the enrollment period were excluded. Therefore, the number of included patients was equal to the number of included THAs in this study.

Patients were categorized into two groups according to the fixation method of THA: with cement versus without cement. The patient demographics and characteristics, including age, sex, underlying diseases (Charlson Comorbidity Index [CCI]), level of medical institution where patients were operated, length of hospital stay, and type of anesthesia, were collected.^20^ Medical institutions were classified into tertiary hospitals (number of beds ≥500), general hospitals (number of beds: 100-499), hospitals (number of beds: 30-99), and clinics (number of beds <30).

### Outcome Measures

The survivorship of THAs with cement and that of THAs without cement were calculated using seven different end points: revision of both the cup and stem, revision of the single component: cup or stem, revision surgery for any reason, PJI, and PPF.

The type of revision was identified using the procedural code of revision (N1711, N3710, N1721, and N3720) and device code of components.

PJIs (T845 and T847) and PPFs (M966) were identified using the diagnostic codes, which were added after the index THA.

### Statistical Analysis

Patient features were compared between cemented and noncemented THA groups using the chi-squared statistic for categorical variables and the Student *t*-test for continuous variables. Univariate and multivariate Cox proportional hazard analyses with noncemented fixation as a reference were performed to evaluate the effect of the fixation method on revisions, PJIs, and PPFs. Hazard ratios (HRs) and the 95% confidence intervals (CIs) adjusted for age and sex were evaluated in the first model. The second model was adjusted for age, sex, and CCI. Survival analysis using the Kaplan-Meier method was performed to estimate the survival rates of the cemented and noncemented THA. All data analyses were performed using R software (version 3.5.3). For all analyses, probability values of 0.05 were considered significant.

As it was a retrospective registry study, approval of this study was exempted by the institutional review board of our hospital with waived informed consent of involved patients.

## Results

### Epidemiology of Cemented Versus Noncemented Total Hip Arthroplasty on Osteonecrosis of the Femoral Head

**Figure 1:**
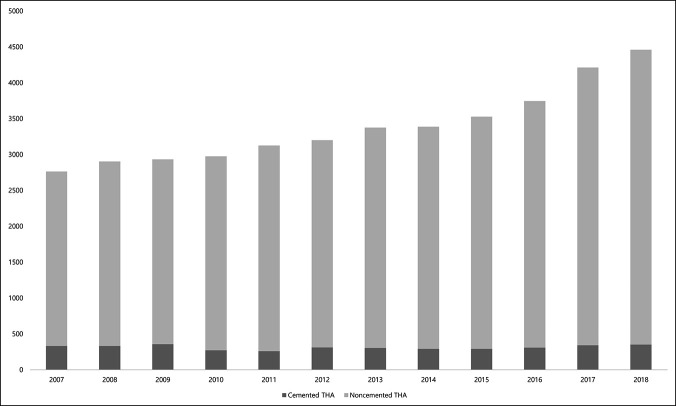
Annual change of the number of cemented vs noncemented THA on patients with ONFH. ONFH = osteonecrosis of the femoral head, THA = total hip arthroplasty

A total of 40,606 patients received THA for ONFH from January 2007 to December 2018. THAs with cemented fixation and noncemented fixation accounted for 3,738 patients (9.2%) and 36,868 patients (90.7%), respectively. Although the number of noncemented THA for ONFH has continuously increased from 2,433 cases in 2007 to 4,111 cases in 2018, the number of cemented THA fluctuated between 259 cases and 355 cases during the same period (Figure 1).

The mean age of patients undergoing THA for ONFH has steadily increased from 54.8 ± 13.5 years in 2007 to 57.1 ± 13.1 years in 2018. The mean age of the cemented fixation group (57.0 ± 15.7 years) was significantly higher than that of the noncemented fixation group (56.2 ± 13.2 years) (*P* = 0.003), with both groups showing male preponderance. Noncemented THAs were more frequently performed in the higher level of medical institutions compared with cemented THAs. General anesthesia for THA accounted for 39% of noncemented THAs but 74% of cemented THAs (*P* < 0.001). Severity of underlying comorbidities represented by mean CCIs was 0.8 ± 1.1 in the noncemented group compared with 0.7 ± 1.1 in the cemented group (*P* < 0.001) (Table 1).

**Table 1:** Demographics of Cemented vs Noncemented THA on ONFH From 2007 to 2018

Variables	Noncemented (n = 36,868)	Cemented (n = 3,738)	*P* value
Age, mean ± SD	56.2 ± 13.2	57.0 ± 15.7	0.003
Sex (male), n (%)	24,632 (66.8)	2,372 (63.5)	<0.001
Level of medical institution			<0.001
Tertiary hospital	11,536 (31.29)	950 (25.41)	
General hospital	12,223 (33.15)	660 (17.66)	
Hospital	12,690 (34.42)	2,092 (55.97)	
Clinics	419 (1.14)	36 (0.96)	
Hospital stay (days)	18.2 ± 10.0	19.8 ± 12.1	<0.001
Type of anesthesia			<0.001
General, n (%)	14,458 (39.26)	2,777 (74.35)	
Regional, n (%)	22,372 (60.74)	958 (25.65)	
Charlson Comorbidity Index	0.8 ± 1.1	0.7 ± 1.1	<0.001
Myocardial infarction, n (%)	234 (0.6)	23 (0.6)	0.887
Congestive heart failure, n (%)	624 (1.7)	72 (1.9)	0.294
Peripheral vascular disease, n (%)	611 (1.7)	73 (2.0)	0.181
Cerebrovascular disease, n (%)	1,624 (4.4)	144 (3.9)	0.115
Dementia, n (%)	91 (0.3)	7 (0.2)	0.480
Chronic pulmonary disease, n (%)	5,261 (14.3)	532 (14.2)	0.950
Rheumatologic disease, n (%)	1,417 (3.8)	103 (2.8)	0.001
Peptic ulcer disease, n (%)	2,941 (8.0)	296 (7.9)	0.900
Mild liver disease, n (%)	4,276 (11.6)	325 (8.7)	<0.001
Diabetes without chronic complication, n (%)	3,104 (8.4)	282 (7.5)	0.065
Diabetes with chronic complication, n (%)	1,165 (3.2)	128 (3.4)	0.380
Hemiplegia or paraplegia, n (%)	140 (0.4)	14 (0.4)	0.961
Renal disease, n (%)	900 (2.4)	51 (1.4)	<0.001
Any malignancy including leukemia and lymphoma, n (%)	1,525 (4.1)	117 (3.1)	0.003
Moderate or severe liver disease, n (%)	132 (0.4)	10 (0.3)	0.372
Metastatic solid tumor, n (%)	61 (0.2)	4 (0.1)	0.394

### Revisions and Complications

Cemented fixation in THAs for ONFH was at higher risk of acetabular cup revision compared with noncemented fixation in both unadjusted analysis and when adjusted for age, sex, and type of medical institutions.

The risk of femoral stem revisions was not associated with the type of fixation. No difference was found for partial revision, which includes only acetabular cup revision and only femoral stem revision between the two groups as well.

Both revisions, which were a simultaneous revision of the acetabular cup and femoral stem, were at notably higher risk in the cemented group.

The risk of any revision, which includes both revisions, cup revision, and stem revision, was notably higher in cemented THA.

Although the risk of PJI was notably higher in the cemented group, the risk of PPF was not different between the two groups (Table 2).

**Table 2:** Cox Proportional Hazard Analysis for the Effect of the Type of Fixation on Revisions and Complications

Outcome		Unadjusted HR (95% CI)	Adjusted HR (95% CI)
Cup revision	Noncemented	1 (reference)	
	Cemented	1.52 (1.13–2.05)	1.40 (1.04–1.89)
Stem revision	Noncemented	1 (reference)	
	Cemented	1.00 (0.69–1.47)	1.07 (0.73–1.57)
Partial revision	Noncemented	1 (reference)	
	Cemented	1.25 (0.99–1.59)	1.23 (0.97–1.57)
Both revisions	Noncemented	1 (reference)	
	Cemented	2.11 (1.68–2.65)	1.92 (1.52–2.41)
Any revision	Noncemented	1 (reference)	
	Cemented	1.52 (1.27–1.81)	1.44 (1.21–1.72)
PJI	Noncemented	1 (reference)	
	Cemented	1.72 (1.41–2.10)	1.66 (1.36–2.04)
PPF	Noncemented	1 (reference)	
	Cemented	0.78 (0.56–1.07)	0.76 (0.55–1.06)

Adjusted for age, sex, and type of medical institution.

CI = confidence interval, HR = hazard ratio, PJI = periprosthetic joint infection, PPF = periprosthetic fracture

### Survivorship

**Figure 2:**
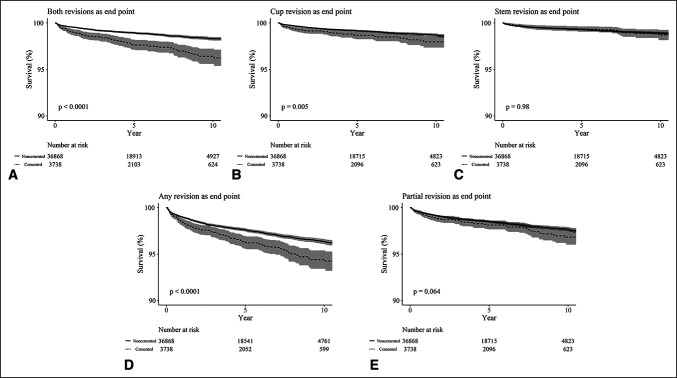
**A,** Kaplan-Meier survival analysis with both revisions as the end point. **B,** Kaplan-Meier survival analysis with cup revision as the end point. **C,** Kaplan-Meier survival analysis with stem revision as the end point. **D,** Kaplan-Meier survival analysis with any revision as the end point. **E,** Kaplan-Meier survival analysis with partial revision as the end point

When implant revision was set as an end point, survival rates at 12 years were superior in noncemented THA compared with cemented THA in both cup and stem revisions (*P* < 0.0001), acetabular cup revision (*P* = 0.005), and any component revision (*P* < 0.0001). However, in terms of femoral stem revision (*P* = 0.98) and partial revision (one component; either cup or stem revision) (*P* = 0.064), there were no significant differences (Table 3 and Figure 2).

**Table 3:** Rate of Survival for Each End Point

Group	Variables	Survival Rate (95% CI)
Noncemented	Both revisions	98.17 (98.06–98.29)
	Cup revision	98.54 (98.43–98.65)
	Stem revision	98.78 (98.68–98.88)
	PJI	97.31 (97.17–97.45)
	PPF	97.63 (97.46–97.80)
	Any revision	96.05 (95.88–96.22)
	Partial revision	97.43 (97.29–97.57)
Cemented	Both revisions	95.91 (95.36–96.47)
	Cup revision	97.96 (97.65–98.28)
	Stem revision	98.70 (98.42–98.98)
	PJI	95.68 (95.20–96.16)
	PPF	98.02 (97.56–98.48)
	Any revision	93.88 (93.25–94.51)
	Partial revision	96.81 (96.40–97.22)

CI = confidence interval, PJI = periprosthetic joint infection, PPF = periprosthetic fracture

**Figure 3:**
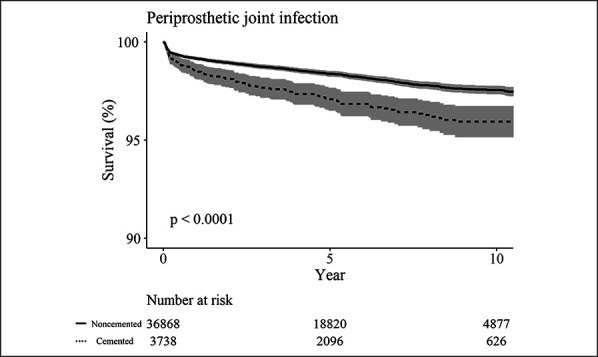
Kaplan-Meier survival analysis with PJI as the end point. PJI = periprosthetic joint infection

The survival rate with PJI as an end point was superior in noncemented THA compared with cemented THA (*P* < 0.0001) (Table 3 and Figure 3).

**Figure 4:**
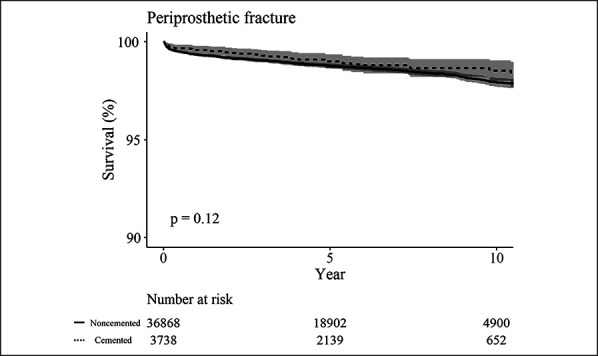
Kaplan-Meier survival analysis with PPF as the end point. PPF = periprosthetic fracture

The survival rate with PPF as an end point was not significantly different between two fixation methods of THA (*P* = 0.12) (Table 3, Figure 4).

## Discussion

From 2007 to 2018, noncemented fixation in THA accounted for 90.7% of the entire THA on ONFH in South Korea. The number of noncemented THA has grown 69%, whereas the number of cemented THA remained similar during the same period. Patients who received cemented THA were notably older, were less male predominant, had less underlying comorbidities, were likely to be operated under general anesthesia, and were treated in lower level medical institutions. Despite the excellent survival rate in both fixation methods, THA with cemented fixation had a notably higher risk of cup revision, both revisions, any revision, and PJI. The risk of PPF was marginally higher in noncemented THA but did not reach statistical significance.

The preference of noncemented THA in patients with ONFH in South Korea is in line with the recent global trends.^12,21-23^ Over 90% of arthroplasty surgeons in the Orthopedic Research Network, which covers 174 hospitals in the United States, responded that they use noncemented femoral stems in at least 75% of the elective THAs.^21^ The proportion of noncemented THAs has increased from 49% in 2001 to 94% in 2012.^23^ In 2013, Bedard et al^14^ reported superior 10-year outcomes of noncemented THA compared with cemented THA on patients with ONFH. Miladi et al^24^ also reported comparable outcomes of noncemented stems at least 7-year follow-up. However, cemented fixation is still favored in osteoporotic patients with femur neck fractures, mainly because of the lower incidence of intraoperative PPF.^7,25^

One of the important factors for considering noncemented fixation over cemented fixation is the bone cement implantation syndrome (BCIS). BCIS is a potentially life-threatening complication related to the implantation of polymethyl methacrylate bone cement. The symptoms include hypoxia, hypotension, or unconsciousness after cementation, which could lead to massive pulmonary embolism and cardiac arrest.^5^ Several studies show the higher short-term mortality rate in cemented hemiarthroplasty compared with the noncemented counterparts.^11,26,27^ Rassir et al^4^ suggested that surgeons should weigh the risk of PPF and BCIS in choosing the fixation method in hemiarthroplasty. In patients with ONFH, who are relatively younger and less osteoporotic than the patients with hip fracture, the risk of PPF seems to be less crucial, which might lead to the higher proportion of noncemented fixation.

Many registry studies have compared the clinical outcomes of THA with cemented and noncemented fixations (Table 4).^6,7,8,9,10,13,15,16,17,18^ The revision rate ranged from 0% to 7.6% in cemented THAs and 0.84% to 14.8% in noncemented THAs. Four of the studies^7,9,10,16^ were conducted on patients with femur neck fracture or osteoporosis. One study included patients who received cemented or noncemented THAs with well-known excellent outcomes regardless of diagnosis. The authors concluded that the overall revision rate did not differ between two groups after 3 months postoperatively.^6^ Wechter et al reported an aseptic revision rate of 3.8% in cemented fixation and 1.6% in noncemented fixation at 20 years. The hazard ratio (HR) for stem revisions was 3.76 (95% confidence interval [CI] 2.01-7.06) in cemented implants, with the noncemented implants as the reference (*P* < 0.0001).^15^ Kelly et al^18^ reported an aseptic revision incidence of 2.3% in hybrid (cemented femoral implant and noncemented acetabular implant) fixation and 2.6% in noncemented fixation (*P* = 0.357), with the adjusted HR of aseptic revision of 0.90 (95% CI 0.73-1.12) in hybrid fixation. In a recently published registry study, Boyle et al compared noncemented, hybrid, and cemented THAs in patients younger than 40 years and those aged between 40 to 55 years. They concluded that revision rates were significantly lower in the hybrid group (HR 0.39, *P* < 0.001) and the noncemented group (HR 0.41, *P* < 0.001) compared with the cemented group in patients aged 40 to 55 years.^17^ In our study, noncemented fixation was superior in terms of cup revision (HR 1.40 [95% CI 1.04-1.89], *P* = 0.027) and both revisions (HR 1.92 [95% CI 1.52-2.41], *P* < 0.001), although no significant difference was found in stem revisions (HR 1.07 [95% CI 0.73-1.57], *P* = 0.737) at 12 years. With the continuous advancements in cementing techniques, especially in the femoral stem insertion, the higher revision rate of cemented THAs seems to be prominently associated with aseptic loosening of the acetabular implant. This assumption is consistent with the previous reports, where acetabular loosening was the main reason for failure in cemented THAs.^17,28^

**Table 4:** Clinical Outcomes of Cemented vs Noncemented THA in Registry Studies

First Author	Country	Year	Database	Diagnosis	Type of Fixation	No. of Hips	Age (Years)	FU (Years)	PPF	Other Complications
Wechter^15^	US	2013	HEJR	OA, ONFH, and others	Cemented	2,179	72	10.5	N/A	Aseptic loosening 3.8% Stem revision 4.9% HR All stem revision 1.63 Stem revision for aseptic loosening 3.76
					Noncemented	4,319	62	5.6	N/A	Aseptic loosening 1.6% Stem revision 3.8%
Tanzer^6^	Canada Australia	2018	AOANJRR	N/A	Cemented	31,635	N/A	13	0.5%	PJI 0.2% Loosening 0.1% Dislocation 0.1% Revision 1.0%
					Noncemented	5,023	N/A	13	1.2%	PJI 0.1% Loosening 0.4% Dislocation 0.1% Revision 2.0%
Yang^10^	China	2019	South China Hip Arthroplasty	Osteoporosis	Cemented	184	71	6.3	1.6%	Loosening 16.8% Revision 7.6% Shaft fracture 3.3%
					Noncemented	182	72	6.3	1.6%	Loosening 26.4% Revision 14.8% Shaft fracture 2.7%
Liu^7^	China	2019	South China Hip Arthroplasty	FNF	Cemented	164	69	6.1	4.9%	Revision 1.8% Loosening 8.5% Dislocation 3% PJI 1.2%
					Noncemented	160	69	6.1	11.9%	Revision 7.5% Loosening 17.5% Dislocation 6.9% PJI 1.3%
Lindberg-Larsen^8^	Denmark	2020	LCDB	OA	Cemented	3,368	79	30 days	0.2%	30-Day mortality 0.2% Dislocations 1.2% In-hospital complications 7.7% Readmission 5.7%
					Noncemented	4,728	76	30 days	1.5%	30-Day mortality 0.3% Dislocations 1.8% In-hospital complications 5.3% Readmission 6.2%
Oh^16^	US	2020	BPCI, CJR	Fracture, elective	Cemented	359	81	N/A	0%	Readmission 8.4% Revision surgery 0% PJI 0% LOS 2.89 days
					Noncemented	1,312	74	N/A	0.3%	Readmission 6.6% Revision surgery 0.84% Dislocation 0.2% Femoral subsidence 0.15% PJI 0.15% LOS 2.35 days
Pedersen^13^	Sweden, Denmark, Norway, and Finland	2021	NARA	OA	Cemented	108,572	69	N/A	N/A	30-Day mortality 0.21% 90-Day mortality 0.41% 30-Day mortality 0.21%
					Noncemented	80,034		N/A	N/A	14-Day mortality 0.08% 30-Day mortality 0.12% 90-Day mortality 0.26%
Heckmann^9^	US	2021	NRD	Displaced FNF	Cemented	4,427	77	N/A	0.07%	Readmission 8.4% Dislocation 0.8% PJI 0.52% Medical complications 6.69%
					Noncemented	13,064	71	N/A	0.7%	Readmission 7.1% Dislocation 2.5% PJI 0.45% Medical complications 4.71%
Boyle^17^	New Zealand	2021	NZJR	OA, dysplasia, ONFH, and others	Cemented	609	49.9	N/A	0.33%	Revision rate/100 component years 1.75
					Hybrid	3,845	49.6	N/A	0.16%	Revision rate/100 component years 0.62 Revision HR 0.39
					Noncemented	11,459	49.2	N/A	0.03%	Revision rate/100 component years 0.70 Revision HR 0.41
Kelly^18^	US	2022	Kaiser Permanente's TJRR	OA	Hybrid (stem fixation cement)	4,539	77	N/A	0.3%	Septic revision 0.8% Aseptic loosening 0.7% Instability 1.1%
					Noncemented	84,291	66	N/A	0.5%	Septic revision 0.8% Aseptic loosening 0.4% Instability 1.1% Adjusted HR Septic revision 1.5 (1.05-2.14) Aseptic revision 0.90 (0.73-1.12) Loosening 2.18 (1.10-4.35) Instability 0.96 (0.70-1.31) PPF 0.41 (0.22-0.76)

HEJR = HealthEast Joint Registry, AOANJRR = Australian Orthopaedic Association National Joint Replacement Registry, LCDB = Lundbeck Foundation Centre for Fast-track THA and TKA database, BPCI = Bundled Payments for Care Improvement, CJR = Comprehensive Care for Joint Replacement, NARA = Nordic Arthroplasty Register Association, NRD = National Readmissions Database, NZJR = New Zealand Joint Registry, TJRR = Total Joint Replacement Registry, FNF = femur neck fracture, OA = osteoarthritis, ONFH = osteonecrosis of the femoral head, N/A = not accessible, FU = follow-up, PPF = periprosthetic fracture, PJI = periprosthetic joint infection, HR = hazard ratio, LOS = length of hospital stay

Some registry studies have favored cemented THAs in terms of a higher incidence of PPF in noncemented THAs.^6,7,8,9,15,16,18^ The cumulative incidence of PPF ranged from 0% to 4.9% in cemented fixations and 0.03% to 11.9% in noncemented fixations. By contrast, results in current study suggested that there was no significant difference in the risk of PPF between cemented (1.98%) and noncemented THAs (2.69%) (*P* = 0.12). This might be related to the features of the included patients in prior studies. An important factor to be considered is the age of the included patients. The mean ages of patients of noncemented THA and cemented THA groups were 56.2 years and 57.0 years, respectively. The mean ages of patients in other registry studies range from 62 years to 81 years. Interestingly, Kelly et al^18^ stratified the risk of revision by age and sex and found that the risk of PFF is higher in noncemented fixation in all age groups, but the difference of PPF risk between the two groups increases from 0.4% in patients younger than 65 years to 0.9% in patients older than 75 years. Therefore, the age discrepancy between the current study and the prior studies might be related to a higher risk of PPF not only in the osteoporotic fracture cohort but also in patients with OA.

In this study, the risk of PJI was notably higher (HR 1.66 [95% CI 1.36 to 2.04]) in THA using cemented fixation. Tanzer et al reported 0.2% of PJI in cemented THAs and 0.1% in noncemented THAs,^6^ and Heckmann et al^9^ reported 0.52% in cemented THAs and 0.45% in noncemented THAs. By contrast, Oh et al.^16^ found 0.15% of PJI in noncemented THAs and none of PJI in cemented cases. In a meta-analysis including eight studies and 84,200 hips, Yoon et al. reported a 0.2% higher incidence of PJI in cemented THAs compared with noncemented THAs.^29^ The difference of PJI risks between cemented and noncemented fixation could be related to longer operation time in cemented THAs for cementing procedures. Moreover, the bone cement itself could be a susceptible factor to infection.^30^ Other relevant factors of the increased PJI risk in cemented fixation are the thermal necrosis of the adjacent bone due to cement polymerization or biofilm development at the cement-bone interface.^31^

This study has several limitations. First, as all events were coded using the operational definition, if the codes were initially entered incorrectly, the outcomes could be biased. However, we combined the diagnostic and operational codes along with the information on admission in formulizing the operational definition to minimize the bias. Second, although the type of articulations of THAs is crucial in long-term survival of the implant, they were not controlled because of the lack of the sufficient identification in HIRA database. All types of acetabular liners and femoral heads are registered in HIRA database, but the specific information is not opened to public. We were unable to acquire the specifics of the bearings and could not perform further analysis with the articulations controlled. Third, this study was performed in patients with ONFH in South Korea. The etiological background of ONFH in East Asia is mainly alcohol related or idiopathic, which is different from the Western countries. The regional and ethnic diversity should be taken into consideration when applying our results. Despite the limitations, this registry study was conducted in a homogenous group of patients with ONFH, using a very thorough database that covers nearly 100% of patients in South Korea.

The noncemented fixation accounted for 91% of THAs on patients with ONFH from 2007 to 2018. Noncemented THAs were superior, especially in acetabular cup revision and PJI. The risk of femoral stem revision or PPF did not show a notable difference between two fixation methods. Noncemented THAs seem to be a more reasonable and effective option in patients with ONFH.
